# Preferences for Firearm Locking Devices and Device Features Among Participants in a Firearm Safety Event

**DOI:** 10.5811/westjem.2019.5.42727

**Published:** 2019-07-01

**Authors:** Joseph A. Simonetti, Cassie Simeona, Chelsie Gallagher, Elizabeth Bennett, Frederick P. Rivara, Ali Rowhani-Rahbar

**Affiliations:** * Rocky Mountain Regional VA Medical Center, Rocky Mountain Mental Illness Research, Education and Clinical Center, Aurora, Colorado; † University of Colorado School of Medicine, Division of Hospital Medicine, Aurora, Colorado; ‡ Seattle Children’s Hospital, Seattle, Washington; § University of Washington, Department of Epidemiology, Seattle, Washington; ¶ University of Washington, Harborview Injury Prevention & Research Center, Seattle, Washington

## Abstract

**Introduction:**

Safe firearm storage is associated with a lower risk of firearm-related injury and death. Although providing firearm locking devices is a key component of firearm safety interventions, little is known about the types and characteristics of devices preferred by firearm users or others who make decisions about firearm storage. The aim of this study was to describe preferences for firearm locking devices and device features among firearm safety event participants.

**Methods:**

We conducted a cross-sectional survey in the State of Washington in 2016 that assessed participants’ preferences for five firearm locking devices (eg, trigger lock) and seven device features (eg, quick access). We categorized respondents (n=401) as adults in households with 1) all firearms locked, 2) at least one unlocked firearm, and 3) no firearms. We analyzed data in 2017.

**Results:**

Device ownership and feature preferences varied substantially but were similar across the three household categories. Of those residing with unlocked firearms, 84% reported they would consider using or definitely use a lock box, whereas 11% reported they would never use a trigger lock. Additionally, of those residing with unlocked firearms, 80% and 89% reported that the ability to lock a firearm while loaded and unlock it quickly were, respectively, “very important” or “absolutely essential.”

**Conclusion:**

Participants had differing preferences for firearm locking devices and device features, although preferences were largely similar across households with locked, unlocked, or no firearms. At least eight in ten participants reported “great importance” regarding the ability to lock a firearm while loaded and unlock it quickly, which is likely related to perceptions about the utility of safely stored firearms for household protection. Designing firearm safety interventions to match the needs and preferences of those who make firearm storage decisions may improve their effectiveness.

## INTRODUCTION

Safe firearm storage (i.e., storing firearms locked and unloaded) is associated with a lower risk of firearm-related suicide as well as firearm-related unintentional injury and death.^1^–^3^ Several interventions have been shown to be effective in promoting safe firearm storage.^4^,^5^ The provision of firearm locking devices appears to be a key component of successful interventions.^4^ However, little is known about preferences for different, commercially-available locking devices, including external locking mechanisms (e.g., trigger, cable, and Life Jacket™ locks) or storage containers in which firearms can be secured (e.g., firearm safes, lock boxes) (Appendix A). A recent community-based firearm safety intervention found that 96% of participants elected to receive a free firearm lock box rather than a trigger lock.^6^ This finding is consistent with a small study among rural Alaskan households in which participants preferred firearm safes instead of trigger locks and were much less likely at follow-up to use trigger locks than safes to store their firearms.^7^

These findings are concerning given that most interventions have relied on distributing cable or trigger locks to promote changes in firearm storage behaviors, largely due to their relatively low cost and ease of distribution.^4^,^5^ Such a “one size fits all” approach may be ineffective in promoting population-level changes in storage practices given the diversity in characteristics of firearm owners, types of firearms owned, and firearm uses.^8^–^10^ A majority of firearm owners in the U.S. report that protection is a primary reason for their firearm ownership.^8^,^10^ Storage preferences (e.g., ease of access) may differ among those owning firearms for hunting or target shooting rather than protection. Aligning intervention characteristics with the needs and preferences of those who make decisions on firearm storage practices is necessary. The aims of this study were to provide a detailed description of preferences for multiple, available firearm locking devices and the first description of preferences for locking device features among firearm safety event participants.

## METHODS

### Study Design

We conducted a cross-sectional survey among participants in two community-based, firearm safety events in the State of Washington in 2016. We included participants who were 18 years or older, spoke English or Spanish, signed a legal release form necessary for event participation, and returned a completed survey. This evaluation was exempted from review by the human subjects divisions of the University of Washington and Seattle Children’s Hospital. We analyzed the data in 2017.

### Firearm Safety Event and Survey Procedure

Events were held at community retail locations where participants received a brief safety message and their choice of a free firearm trigger lock or lock box. Additional details have been published previously.^6^ Prior to event participation, participants completed a voluntary, 23-item survey assessing firearm storage practices, reported and considered use of specific firearm storage devices, and perceived importance of specific device features. Pictures of specific storage devices and their approximate costs were presented to participants using a visual placard that showed a trigger lock ($5–15), cable lock ($5–15), Life Jacket™ ($20–30), lock box ($20–100), and a firearm cabinet/safe ($100 or more) (Appendix A).

### Statistical Analysis

We classified respondents into three categories, namely those who reported the following: 1) all household firearms were stored locked; 2) at least one household firearm was unlocked; and 3) no firearms were kept in their homes. We described device ownership and use across these groups and compared device feature preferences between them using chi-squared tests.

## RESULTS

Of 583 participants, 401 returned completed surveys (68.8% response proportion). Demographic characteristics and storage practices are shown in the supplemental table. Prevalence of device ownership and reported device use or consideration of use was similar across the three groups (Table 1). A greater proportion of respondents within each household category reported that they would never use a trigger lock (4.2–8.9%), cable lock (7.7–11.4%), or Life Jacket™ (7.6–14.4%) compared to a lock box (0.6–2.8%) or firearm safe (0–4.3%). Large proportions within each household category reported they would consider using, or definitely use, each of the devices if they owned it (51.9–85.5%). Those reporting at least one unlocked household firearm were most likely to report that they would consider using, or definitely use, a lock box if owned (84.0%), followed by the Life Jacket™ (82.4%), trigger lock (78.6%), firearm safe (76.5%), and cable lock (68.9%).

Preferences for device features were generally similar across the three groups (Table 2). Eighty percent and 89% reported that the ability to lock a firearm while loaded and to unlock it quickly, respectively, was “*very important”* or “*absolutely essential*,” whereas 12% and 26% reported that device appearance and device cost of less than $15, respectively, was “*very important”* or “*absolutely essential*.” Of those who responded to the survey, 80–90.2% reported that ease of transfer between vehicle and home, ability to use the device on both handguns and long guns, and recommendation of the device by a law enforcement agency or firearm advocacy group were at least “*moderately important*.”

## DISCUSSION

In this study we found that firearm safety event participants had differing preferences for firearm locking devices and device features, although preferences were largely similar among households with locked, unlocked, or no firearms. To our knowledge, no prior work has assessed preferences for firearm locking devices or device features in such detail.

These findings have important implications for safe firearm storage interventions. Most interventions have focused on distributing single devices (usually cable or trigger locks).^4^,^5^ Having only offered trigger locks during these events would not have addressed the 8% of those in firearm households who reported they would never use this device and the 4% who already owned but did not use them. There are many variations in what storage options might work best for gun owners given the variety of firearms available and reasons for ownership and use. Participant-centered interventions designed to address this variation are likely to be more effective.

At least eight in ten participants reported “great importance” regarding the ability to lock a firearm while loaded and unlock it quickly. This is likely related to the fact that two-thirds of firearm owners keep firearms for protection and perceptions that the time required to unlock a firearm may interfere with that purpose.^8^,^10^ Such strong preferences should be considered in deciding what types of devices are distributed in safety device promotion interventions. However, those who develop interventions with the aim of preventing firearm suicides must also consider that delaying access to a firearm during an emotional crisis is precisely one of the purposes of the locking device. In this scenario, there may be a role for developing communication strategies to be incorporated into firearm safety interventions that address risk misperceptions (e.g., balancing the risk of harm to oneself and household members vs harm from others).

## LIMITATIONS

This study was conducted among event participants in the State of Washington, and specific findings on device preferences may not apply elsewhere. What is generalizable, however, is that gun owners have preferences that must be addressed if they are to be expected to use a product – a concept that has yet to be applied broadly to firearm safety interventions. A small proportion who identified themselves as living in non-firearm owning households also reported owning and using firearm safety devices. This finding can be explained if respondents were reluctant to report firearm ownership, completed this item in error, used devices on firearms stored outside the home, if devices were used to store non-firearm items (e.g., valuables stored in locked safe), or if they intended to give the storage device to someone else.

## CONCLUSION

This study provides the first detailed insights into preferences for firearm safety devices among adults in both firearm and non-firearm owning households. Determining whether the consideration of these preferences in the design of firearm safety interventions improves their effectiveness is warranted.

## Supplementary Information





## Figures and Tables

**Table 1:** Device use and considered use by household firearm ownership and storage practices (n=401)*^

	Firearm-owning household, all firearms locked					Firearm-owning household, at least one unlocked firearm					Non-firearm owning household				

	n=185					n=141					n=75				

	Would never use if owned	Would consider using if owned	Would definitely use if owned	Owns and uses	Owns but does not use	Would never use if owned	Would consider using if owned	Would definitely use if owned	Owns and uses	Owns but does not use	Would never use if owned	Would consider using if owned	Would definitely use if owned	Owns and uses	Owns but does not use
Trigger lock	13 (7.2%)	19 (10.5%)	100 (55.3%)	39 (21.6%)	10 (5.5%)	12 (8.9%)	38 (28.2%)	68 (50.4%)	14 (10.4%)	3 (2.2%)	3 (4.2%)	10 (13.9%)	49 (68.1%)	7 (9.7%)	3 (4.2%)
Cable lock	14 (7.7%)	33 (18.2%)	79 (43.7%)	43 (23.8%)	12 (6.6%)	15 (11.1%)	35 (25.9%)	58 (43.0%)	18 (13.3%)	9 (6.7%)	8 (11.4%)	13 (18.6%)	40 (57.1%)	6 (8.6%)	3 (4.3%)
Life Jacket™	13 (7.6%)	60 (34.9%)	87 (50.6%)	10 (5.8%)	2 (1.2%)	18 (14.4%)	39 (31.2%)	64 (51.2%)	4 (3.2%)	0 (0%)	8 (11.6%)	13 (18.8%)	45 (65.2%)	2 (2.9%)	1 (1.5%)
Lock box	1 (0.6%)	11 (6.0%)	112 (61.5%)	40 (22.0%)	18 (9.9%)	3 (2.2%)	9 (6.5%)	107 (77.5%)	12 (8.7%)	7 (5.1%)	2 (2.8%)	5 (6.9%)	54 (75.0%)	7 (9.7%)	4 (5.6%)
Firearm safe	0 (0%)	11 (6.0%)	84 (45.9%)	61 (33.3%)	27 (14.8%)	3 (2.2%)	9 (6.6%)	95 (69.9%)	22 (16.2%)	7 (5.2%)	3 (4.3%)	5 (7.1%)	50 (71.4%)	9 (12.9%)	3 (4.3%)

* Cell data are frequencies and corresponding row percentages. Row frequencies may not sum to totals due to missing data.

^ Participants were able to report ownership of more than one device.

**Table 2:** Importance of specific firearm locking device features by household firearm ownership and storage practices (n=401).* Question stem: “Please tell us how important each of these features is to you in a gun locking device.

	Total	Firearm-owning household, all firearms locked	Firearm-owning household, at least one unlocked firearm	Non-firearm owning household	p value

	n=401	n=185	n=141	n=75	
Can unlock the device quickly					0.67
Not at all/little importance	9 (2.3%)	3 (1.6%)	5 (3.6%)	1 (1.3%)	
Moderate importance	34 (8.5%)	14 (7.6%)	12 (8.6%)	8 (10.7%)	
Very important	150 (37.5%)	66 (35.7%)	54 (38.6%)	30 (40.0%)	
Absolutely essential	207 (51.8%)	102 (55.1%)	69 (49.3%)	36 (48.0%)	
Device costs less than 15 United States dollars					0.09
Not at all/little importance	164 (41.4%)	81 (44.3%)	55 (39.6%)	28 (37.8%)	
Moderate importance	128 (32.3%)	56 (30.6%)	51 (36.7%)	21 (28.4%)	
Very important	69 (17.4%)	24 (13.1%)	25 (18.0%)	20 (27.0%)	
Absolutely essential	35 (8.8%	22 (12.0%)	8 (5.8%)	5 (6.8%)	
Can lock firearm while it is loaded					0.52
Not at all/little importance	28 (7.0%)	15 (8.2%)	8 (5.7%)	5 (6.8%)	
Moderate importance	51 (12.8%)	27 (14.7%)	15 (10.7%)	9 (12.2%)	
Very important	131 (32.9%)	51 (27.7%)	57 (40.7%)	23 (31.1%)	
Absolutely essential	188 (47.2%)	91 (49.5%)	60 (42.9%)	37 (50.0%)	
Appearance of locking device					0.03
Not at all/little importance	283 (71.1%)	137 (74.9%)	105 (75.0%)	41 (54.7%)	
Moderate importance	67 (16.8%)	30 (16.4%)	20 (14.3%)	17 (22.7%)	
Very important	32 (8.0%)	9 (4.9%)	10 (7.1%)	13 (17.3%)	
Absolutely essential	16 (4.0%)	7 (3.8%)	5 (3.6%)	4 (5.3%)	
Easy transfer between vehicle and home					0.66
Not at all/little importance	39 (9.8%)	21 (11.4%)	14 (10.0%)	4 (5.3%)	
Moderate importance	94 (23.6%)	38 (20.7%)	33 (23.6%)	23 (30.7%)	
Very important	156 (39.1%)	70 (38.0%)	57 (40.7%)	29 (38.7%)	
Absolutely essential	110 (27.6%)	55 (29.9%)	36 (25.7%)	19 (25.3%)	
Can use on both long guns and handguns					0.18
Not at all/little importance	79 (19.8%)	29 (15.7%)	37 (26.4%)	13 (17.3%)	
Moderate importance	126 (31.5%)	61 (33.0%)	46 (32.9%)	19 (25.3%)	
Very important	114 (28.5%)	52 (28.1%)	37 (26.4%)	25 (33.3%)	
Absolutely essential	81 (20.3%)	43 (23.2%)	20 (14.3%)	18 (24.0%)	
Device recommended by law enforcement agency or firearm advocacy group					0.24
Not at all / little importance	50 (12.5%)	16 (8.7%)	24 (17.3%)	10 (13.3%)	
Moderate importance	105 (26.3%)	49 (26.5%)	38 (27.3%)	18 (24.0%)	
Very important	143 (35.8%)	63 (34.1%)	50 (36.0%)	30 (40.0%)	
Absolutely essential	101 (25.3%)	57 (30.8%)	27 (19.4%)	17 (22.7%)	

* Rows may not sum up to total population due to missing data.
