# Supplementary material for: Preferences for Firearm Locking Devices and Device Features Among Participants in a Firearm Safety Event
Source: West J Emerg Med. 2019 Jul 1;20(4):552–6. doi: 10.5811/westjem.2019.5.42727 (PMC6625681; doi:10.5811/westjem.2019.5.42727)
Supplement: Supplementary file 1 [file wjem-20-552-s001.docx]

**Appendix A.** Descriptions of firearm safety devices.

**Cable Lock:** Placed through the gun chamber or magazine well to prevent loading and firing. It is unlocked with a combination or key.


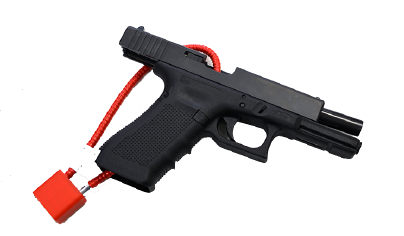


**Trigger Lock:** Goes through the trigger guard behind the trigger, preventing the trigger from being pulled. It can be unlocked with a keypad, combination, or key.


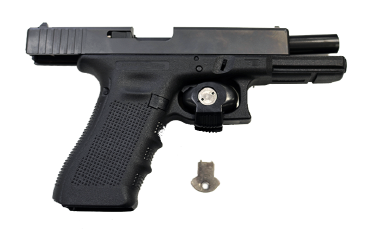


**Life Jacket (not pictured):** An enclosure that envelops the firearm, preventing access to any operational parts of the gun. It can be unlocked with a key.

**Lock Box:** Small safes that are locked using a key, combination lock, digital keypad, or biometric sensor (eg, fingerprint reader).


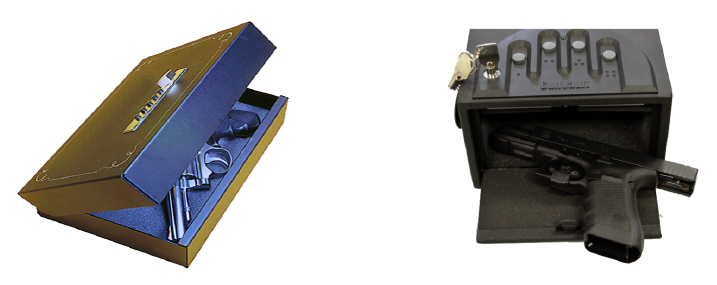


**Firearm Safe/Cabinet:** Firearm safes vary in size and level of protection with some large enough to store rifles and shotguns as well as handguns. Safes may be locked with a key, combination lock, or digital keypad.


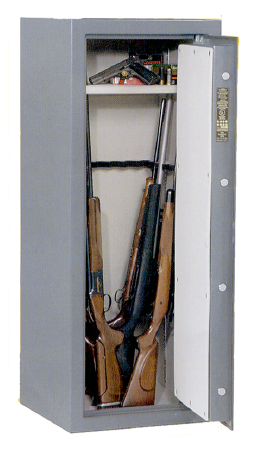


**Footnote.** Images and descriptions for cable lock, trigger lock, lockbox, and firearm safe from: Ad Council and Department of Public Health, Seattle and King County, Washington; Personal Firearms, Programs that Promote Safe Storage and Research on their Effectiveness. United States Government Accountability Office, Report to Congresional Requestors. GAO-17-665. September 2017.

Description of Life Jacket device obtained from www.mseworldwide.com.
