# Supplementary material for: Preferences for Firearm Locking Devices and Device Features Among Participants in a Firearm Safety Event
Source: West J Emerg Med. 2019 Jul 1;20(4):552–6. doi: 10.5811/westjem.2019.5.42727 (PMC6625681; doi:10.5811/westjem.2019.5.42727)
Supplement: Supplementary file 2 [file wjem-20-552-s002.docx]

| **Supplemental Table 1.** Participant and household characteristics and firearm storage practices (n=401) | |
| --- | --- |
|  | *n (%)* |
| Age group, years (n=377) |  |
| 18-25 | 66 (17.5%) |
| 26-45 | 171 (45.4%) |
| 46-65 | 117 (31.0%) |
| 66+ | 23 (6.1%) |
| Preferred language (n=401) |  |
| English | 394 (98.3%) |
| Spanish | 7 (1.8%) |
| Female gender (n=389) | 160 (41.1%) |
| Race/ethnicity (n=393) |  |
| Non-Hispanic White | 232 (59.0%) |
| Non-Hispanic Black | 4 (1.0%) |
| Non-Hispanic Asian | 13 (3.3%) |
| Non-Hispanic AI/AN | 13 (3.3%) |
| Non-Hispanic NH/PI | 7 (1.8%) |
| Hispanic/Latino | 107 (27.2%) |
| Other* | 20 (5.1%) |
| Children in household (n=401) |  |
| Young (>0-10 years) | 104 (25.9%) |
| Adolescent (11-18 years) | 64 (16.0) |
| Both young & adolescent | 41 (10.2%) |
| Any under 18 years | 209 (52.1%) |
| Lives with spouse/significant other (n=401) | 287 (71.6%) |
| Lives alone (n=401) | 38 (9.5%) |
| Military Service (n=392) |  |
| Active | 7 (1.8%) |
| Veteran | 48 (12.3%) |
| Firearms in household (n=401) | 326 (81.3%) |
| **Firearm storage practices** (n=326)** |  |
| All firearms locked | 185 (56.8%) |
| All firearms unloaded | 189 (58.0%) |
| *AI, American Indian; AN, Alaskan Native; NH, Native Hawaiian; PI, Pacific Islander.*  * includes multi-race/multi-ethnicity  ** limited to firearm owners | |
